# Molecular, Genetic, and Biochemical Characterization of OXA-484 Carbapenemase, a Difficult-to-Detect R214G Variant of OXA-181

**DOI:** 10.3390/microorganisms12071391

**Published:** 2024-07-09

**Authors:** Camille Gonzalez, Saoussen Oueslati, Mariam Rima, Réva Nermont, Laurent Dortet, Katie L. Hopkins, Bogdan I. Iorga, Rémy A. Bonnin, Thierry Naas

**Affiliations:** 1 Team “Resist” UMR1184 “Immunology of Viral, Auto-Immune, Hematological and Bacterial Diseases (IMVA-HB)”, Faculty of Medicine, University Paris-Saclay, INSERM, CEA, 94270 Le Kremlin-Bicêtre, France; gonzalezcamille0405@gmail.com (C.G.); oueslati.saoussen@gmail.com (S.O.); mariamrima6@gmail.com (M.R.); revanermont@gmail.com (R.N.); laurent.dortet@aphp.fr (L.D.); remy.bonnin@universite-paris-saclay.fr (R.A.B.); 2 Bacteriology-Hygiene Unit, Assistance Publique-Hôpitaux de Paris, Bicêtre Hospital, 94270 Le Kremlin-Bicêtre, France; 3 French National Reference Center for Antibiotic Resistance, Carbapenemase-Producing Enterobacteriaceae, 94270 Le Kremlin-Bicêtre, France; 4 Antimicrobial Resistance and Healthcare Associated Infections (AMRHAI) Reference Unit, HCAI, Fungal, AMR, AMU and Sepsis Division, UK Health Security Agency, London NW9 5EQ, UK; katie.hopkins@ukhsa.gov.uk; 5 Institut de Chimie des Substances Naturelles, Université Paris-Saclay, CNRS, 91190 Gif-sur-Yvette, France; bogdan.iorga@cnrs.fr

**Keywords:** oxacillinase, carbapenemase, OXA-181, antibiotic resistance, beta-lactamase, detection

## Abstract

OXA-244, an R214G variant of OXA-48, is silently spreading worldwide likely because of difficulties in detection using classical screening media. Here, we characterized two clinical isolates of *Escherichia coli* and *Citrobacter youngae* that displayed reduced susceptibility to carbapenems but were lacking significant carbapenemase activity as revealed by negative Carba NP test results. However, positive test results were seen for OXA-48-like enzymes by lateral flow immunoassays. WGS revealed the presence of a *bla*OXA-181-like gene that codes for OXA-484, an R214G variant of OXA-181. *Bla*OXA-484 gene was located on a 58.4-kb IncP1-like plasmid (pN-OXA-484), that upon transfer into *E. coli* HB4 with impaired permeability, conferred carbapenem and temocillin resistance (MICs > 32 mg/L). *E. coli* TOP10 (pTOPO-OXA-484) revealed reduced MICs in most substrates as compared to *E. coli* TOP10 (pTOPO-OXA-181), especially for imipenem (0.25 mg/L versus 0.75 mg/L) and temocillin (16 mg/L versus 1028 mg/L). Catalytic efficiencies of OXA-484 were reduced as compared to OXA-181 for most ß-lactams including imipenem and temocillin with 27.5- and 21.7-fold reduction, respectively. Molecular modeling confirmed that the salt bridges between R214, D159, and the R1 substituent’s carboxylate group of temocillin were not possible with G214 in OXA-484, explaining the reduced affinity for temocillin. In addition, changes in active site’s water network may explain the decrease in hydrolysis rate of carbapenems. OXA-484 has weak imipenem and temocillin hydrolytic activities, which may lead to silent spread due to underdetection using selective screening media or biochemical imipenem hydrolysis confirmatory tests.

## 1. Introduction

After initial description in Turkey, OXA-48 carbapenemase has rapidly spread in countries of the Mediterranean rim, Middle East, Europe, and India and is now described worldwide, thus becoming a major global health threat [1,2]. Unlike other clinically relevant carbapenemases in Enterobacterales of class A (KPC type) or B (NDM, VIM, IMP), OXA-48 hydrolyzes penicillins including temocillin, narrow-spectrum cephalosporins, and carbapenems at a low rate but spares expanded-spectrum cephalosporins (ESCs), e.g., ceftazidime and cefepime [3,4]. More than 60 variants of OXA-48 have been reported since its initial discovery. They differ from OXA-48 by amino acid (AA) substitutions or deletions (http://bldb.eu/BLDB.php?class=D#OXA, accessed on 1 July 2024), mostly located in the β5-β6 loop [5]. OXA-181 that differs from OXA-48 by four amino-acid substitutions (T103A, N110D, E169Q and S171A) is the second most prevalent OXA-48 variant and exhibits similar ß-lactam hydrolyzing activity as OXA-48 [1,2]. Some variants of both enzymes, such as OXA-244 (R214G of OXA-48) or OXA-232 (R214S of OXA-181), have been described to confer lower MICs for carbapenems and temocillin as compared to OXA-48/OXA-181-producers [6,7]. Recently, OXA-484, an R214G variant of OXA-181 initially described in the UK in *K. pneumoniae*, has since been described in *E. coli* isolates from Germany, UK, Switzerland, South Africa, China, and Ireland [8,9,10,11,12,13,14,15]. In most of these cases, the *bla*_OXA-484_ gene was carried on a 51kb IncX3 plasmid present in *E. coli* ST410 isolates.

Substitutions at position R214 in the β5-β6 loop are particularly interesting, as they significantly impair carbapenem and temocillin hydrolyzing activity as compared to their parental enzymes [6,7]. As temocillin and carbapenems are present in several screening media used for the detection of CPEs, reduced hydrolysis of these molecules can result in difficulties in the detection of these variants [16,17,18]. Because of the difficulties of detection, R214 mutants have the potential to silently spread [1,16,19,20].

Here, we investigated the effect of the R214G substitution on OXA-181 hydrolytic activities and compared them to OXA-244 and OXA-232. In addition, we analyzed the genetic environment of *bla*_OXA-484_ genes in different Enterobacterales from France, UK, and Germany. Finally, using molecular modeling based on the X-ray structure of OXA-181, we were able to propose an explanation for the reduced carbapenem and temocillin hydrolysis.

## 2. Materials and Methods

### 2.1. Bacterial Strains

*E. coli* 172D10 and *C. youngae* 173G7 isolates were sent to the French National Reference Center (F-NRC) for carbapenem-resistant Enterobacterales in 2019 for further characterization as they displayed reduced susceptibility to carbapenems. The isolates were identified using MALDI-TOF (Biotyper, Bruker Daltonics, Hambourg, Germany). *E. coli* TOP10 (Invitrogen, Saint-Aubin, France) and *E. coli* BL21 (DE3) (Novagen, VWR International, Fontenay-sous-Bois, France) were used for cloning and expression experiments, respectively. Azide-resistant *E. coli* J53 was used for conjugation assays. Plasmids of *E. coli* NCTC 50192 were used as plasmid size markers (ca. 154, 66, 48, and 7 kb) [21].

OXA-48-producing *K. pneumoniae* strain 11978 and CTX-M-15-producing *K. pneumoniae* 3C3 were used as positive and negative controls for CarbaNP and lateral flow immunoassay (LFIA), respectively [3,22].

### 2.2. Antimicrobial Agents, Susceptibility Testing, and Microbiological Techniques

Antimicrobial susceptibilities were determined by disk diffusion on Mueller–Hinton agar (Bio-Rad, Marnes-La-Coquette, France) and interpreted according to the EUCAST breakpoints, updated in 2023 (http://www.eucast.org, accessed on 1 July 2024). Minimal inhibitory concentration (MIC) values were determined using the Etest technique for ß-lactam antibiotics (BioMérieux, Paris, France) and broth microdilution for colistin (Sensititre, Thermofisher, Grenoble, France). The ability of *E. coli* 172D10 and *C. youngae* 173G7 to grow on chromID^®^ CARBA SMART (BioMérieux, Marcy-L-Etoile, France) was evaluated by plating 10^3^ CFU of the bacteria. The ChromID Carba Smart, one of the most frequently used media for CPE screening, is a biplate containing, on one side, a carbapenem (allowing growth of most CPEs except some OXA-48-like producers) and, on the other side, temocillin (allowing, specifically, the growth of most OXA-48-like producers) [16,17]. Antibiotics were purchased from Sigma (Saint-Quentin-Fallavier, France), except temocillin (Eumedica, Brussels, Belgium).

Carbapenemase activity was investigated using the Carba NP test [22], and carbapenemases were sought using lateral flow immunoassay (LFIA) NG-Test Carba 5 [23] (NG Biotech, Rennes, France) as recommended by the manufacturer.

### 2.3. PCR, Cloning Experiments, and DNA Sequencing

Using primers preOXA-48A (5′-TATATTGCATTAAGCAAGGG-3′) and preOXA-48B (5′-CACACAAATACGCGCTAACC-3′), a 847-bp fragment containing the entire *bla*OXA-484 gene (798 bp) was amplified and cloned into the pCR^®^-Blunt II-TOPO^®^ plasmid (Invitrogen, Illkirch, France), and the resulting plasmid pTOPO-*bla*_OXA-484_ was subsequently electroporated into *E. coli* TOP10 cells, as previously described [6,7]. Recombinant pTOPO-*bla*_OXA-484_ was selected on kanamycin-containing (50 µg/mL) trypticase soy agar (TSA) plates. The recombinant plasmids pTOPO-*bla*_OXA-232_, pTOPO-*bla*_OXA-48_, pTOPO-*bla*_OXA-181_, and pTOPO-*bla*_OXA-244_ were obtained from previous studies [6,7].

The *bla*_OXA-484_ gene fragment corresponding to the mature β-lactamase (from AA20-265) was cloned into the expression vector pET41b (+) (Novagen, VWR International, Fontenay-sous-Bois, France) and transformed into the chemically competent *E. coli* strain BL21 (DE3), as previously described [6,7].

Recombinant plasmids were extracted and sequenced with an automatic sequencer (ABI Prism 3100; Applied Biosystems, Les Ulis, France) as previously described [6,7]. The nucleotide sequences were analyzed using software available at the National Center for Biotechnology Information website (http://www.ncbi.nlm.nih.gov, accessed on 1 July 2024).

### 2.4. Whole Genome Sequencing (WGS)

Total DNA was extracted from colonies using the Ultraclean Microbial DNA Isolation Kit (MO BIO Laboratories, Carlsbad, CA, USA) following the manufacturer’s instructions. The DNA concentration and purity were quantified by a Qubit^®^ 2.0 Fluorometer using the dsDNA HS and/or BR assay kit (Life technologies, Carlsbad, CA, USA). The DNA library was prepared using the Nextera XT-v3 kit (Illumina, San Diego, CA, USA) according to the manufacturer’s instructions and, then, run on a Miseq (Illumina) generating paired-end 300-bp reads. De novo assembly was performed by CLC Genomics Workbench v 12.0 (Qiagen, Hilden, Germany) after quality trimming (Qs ≥ 20) with word size 34. The acquired antimicrobial resistance genes, the multilocus sequence typing (MLST) profile, the virulence genes, and the different plasmid incompatibility groups were identified by uploading the assembled genomes to the Resfinder v4.0 (https://cge.food.dtu.dk/services/ResFinder/, accessed on 1 July 2024), MLST v2.0 (https://cge.food.dtu.dk/services/MLST/, accessed on 1 July 2024), VirulenceFinder 2.0 (https://cge.food.dtu.dk/services/VirulenceFinder/, accessed on 1 July 2024), and PlasmidFinder 2.1 (https://cge.food.dtu.dk/services/PlasmidFinder/, accessed on 1 July 2024) servers, respectively [24,25,26,27].

### 2.5. Plasmid Characterization and Conjugation Assays

Plasmid DNA of *E. coli* 172D10, extracted using the Kieser method [28], was electroporated into the *E. coli* TOP10 strain. Transformants growing on TSA plates containing 100 µg/mL ampicillin were screened by PCR as previously described [6,7]. Plasmids extracted from parental strains and transformants were subsequently analyzed on 0.7% agarose gel stained with ethidium bromide. Filter mating-out assays between *E. coli* 172D10 and azide resistant *E. coli* J53 as the recipient were performed as previously described [29]. Transconjugants were selected with 100 µg/mL ampicillin and 100 µg/mL azide.

### 2.6. β-Lactamase Purification and Steady-State Kinetic Parameters

An overnight culture of *E. coli* strain BL21 (DE3) harboring pET41b-OXA-484 was used to inoculate 2 L of LB broth containing 50 mg/L kanamycin. Th expression and purification of OXA-484 were carried out as previously described [29]. The protein concentrations were determined by measuring the OD at 280 nm and with the extinction coefficients obtained from the ProtParam tool (https://web.expasy.org/protparam/, accessed on 1 July 2024).

Steady-state kinetic parameters of purified OXA-484 were determined at 30 °C in 100 mM sodium phosphate buffer (pH 7.0). The kcat and Km values were determined by analyzing hydrolysis of β-lactams under initial-rate conditions with an ULTROSPEC 2000 model UV spectrophotometer (Amersham Pharmacia Biotech, Amersham, UK) using the Eadie–Hofstee plot of the Michaelis–Menten equation, as previously described [3,30]. The β-lactams were purchased from Sigma–Aldrich (Saint-Quentin-Fallavier, France).

### 2.7. Molecular Modeling

The effect of the R214G mutation in OXA-181, resulting in OXA-484, was evaluated by molecular modeling. The Dunbrack rotamer library (swapaa command), which is part of the UCSF Chimera software v 1.17.3 [31,32] and which predicts the conformation of the amino acid sidechain based on the global energy minimum of the protein, was used to generate in silico the R214G substitution based on the OXA-181 structure (PDB code 5OE0). Interatomic clashes were identified based on VDW radii [33] using UCSF Chimera software [31,32]. Corina 3.60 (Molecular Networks GmbH, Erlangen, Germany) was used to generate three-dimensional structures of the β-lactam ligands. Molecular docking calculations were performed using Gold (Cambridge Crystallographic Data Centre, Cambridge, UK) [34] and the GoldScore scoring function. The binding site, defined as a 20 Å radius sphere, was centered on the OG oxygen atom of Ser70. All other parameters had default values. The receptor–ligand complex images were produced using UCSF Chimera [32].

### 2.8. Nucleic Acid Sequences

Genomes of *E. coli* 172D10 and *C. youngae* 173G7 were deposited under GenBank accession numbers JBAGCG000000000 and JBAGCF000000000, respectively (bioproject PRJNA1075029). Genomes of the three UK isolates had been submitted to NCBI as part of the bioproject PRJNA788733 (biosamples SAMN24019010, SAMN24297355, SAMN24020527). The sequence of the *E. coli* EC-JS316 was retrieved from GenBank under accession number CP058621.

## 3. Results

### 3.1. Antimicrobial Susceptibility Testing and Carbapenemase Confirmation Tests

Bacterial identification of *E. coli* 172D10 and *C. youngae* 173G7 isolates was confirmed by MALDI-TOF upon reception at the F-NRC. Disk diffusion antibiograms revealed that both isolates were resistant to amoxycillin, ticarcillin, piperacillin, amoxicillin/clavulanic acid, ticarcillin/clavulanic acid, piperacillin/tazobactam, and temocillin, of reduced susceptibility to carbapenems, and remained susceptible to all other ß-lactams tested, including cefiderocol and ceftazidime/avibactam. In addition, both isolates were susceptible to all the other tested antibiotic families, e.g., aminoglycosides, fluoroquinolones, chloramphenicol, tigecyclines, and colistin (confirmed by MIC testing [MIC = 0.25 mg/L]), except to rifampin and to a sulfonamide/trimethoprim association.

Biochemical carbapenemase confirmatory tests based on imipenem hydrolysis (homemade Carba NP [22]) gave repeatedly negative results with *E. coli* 172D10 and inconsistent results with *C. youngae* 173G7, although the resistance phenotype was compatible with the production of an OXA-48-like carbapenemase [1]. The LFIA NG-Test CARBA 5 (NG Biotech) confirmed the presence of an OXA-48-like enzyme in both isolates [23] (Table 1). Finally, as these OXA-48-like producing isolates displayed reduced susceptibility to temocillin and carbapenems, the ability to grow on standard chromogenic and selective media, such as ChromID^®^ CARBA SMART, was tested. Using an inoculum of 10^3^ CFU, both isolates failed to grow on either sides of the plate, suggesting an OXA-244-like variant with lower carbapenem and temocillin hydrolytic activities [16,18]. PCR/sequencing results revealed the presence of *bla*_OXA-484_ gene, a single nucleotide derivative of *bla*_OXA-181_ gene, resulting in a single amino acid substitution, R214G.

MIC values of *E. coli* TOP10 (pTOPO-OXA-484) were compared to those of *E. coli* TOP10 harboring pTOPO-OXA-48, -OXA-244, -OXA-181, and -OXA-232. The R214G substitution irrespective of the OXA-48-like backbone (OXA-48 and OXA-181) resulted in similar MICs for all ß-lactams tested. Reduced MICs for temocillin and imipenem were observed and were similar to those observed for OXA-232, an R214S OXA-181 derivative, and to an in vitro-generated S214G mutant of OXA-232 (Table 1) [6].

Overall, MIC values for ampicillin and cephalothin were not affected, while those of carbapenems (meropenem and ertapenem) varied in the same way as for imipenem. MIC values of *E. coli* HB4 expressing the natural plasmid pN-OXA-484 revealed increased MICs (>32 mg/L for carbapenems) (Table 1), suggesting that these enzymes may confer carbapenem resistance when expressed in a bacterium with impaired outer membrane permeability.

### 3.2. Genomic Features of OXA-484 Producers

The genomes of *E. coli* 172D10 and *C. youngae* 173G7 were determined. Only contigs bigger than 500-bp were retained for further analysis. The genomes were estimated to be 5,462,546 bp and 4,744,353 bp in size, respectively, with a mean sequencing coverage of over 130×. *E. coli* 172D10 and *C. youngae* 173G7 belonged to ST69 and ST491, respectively.

Acquired resistance genes and chromosomal point mutations involved in resistance were further sought. Two acquired β-lactamases, *bla*_TEM-1_, and *bla*_OXA-484_ genes were identified in *E. coli* 172D10, while in *C. youngae* 173G7 ST491, only *bla*_OXA-484_ was present in addition to the chromosome-encoded *bla*_CMY-157_ gene (Table 2). In addition, an *aadA1* gene, conferring resistance to spectinomycin and streptomycin, two sulfonamide resistance genes, *sul1* and *sul2*, *tet*(A), *mdf*(A), and two dihydrofolate reductase genes, *dfrA14* and *dfrA17,* were also present in *E. coli* 172D10, while in *C. youngae* ST491 *aadA1*, *aph(6)-Id*, *sul1,* and three macrolide resistance genes were identified: *ere*(A), *mdf*(A), and *mph*(A). No mutations were identified in topoisomerase genes known to confer resistance to fluoroquinolones in Gram-negative rods. Besides the *bla*_OXA-484_ gene, only a few shared AMR determinants were observed with the isolates from UK and Germany (Table 2). VirulenceFinder identified the *nlpl* gene encoding the lipoprotein NlpI precursor responsible for epithelial cell adhesion and invasion in Uropathogenic *E. coli* (UPEC) isolates [35].

### 3.3. Genetic Support and Environment of bla_OXA-484_ Gene

**Figure 1:**
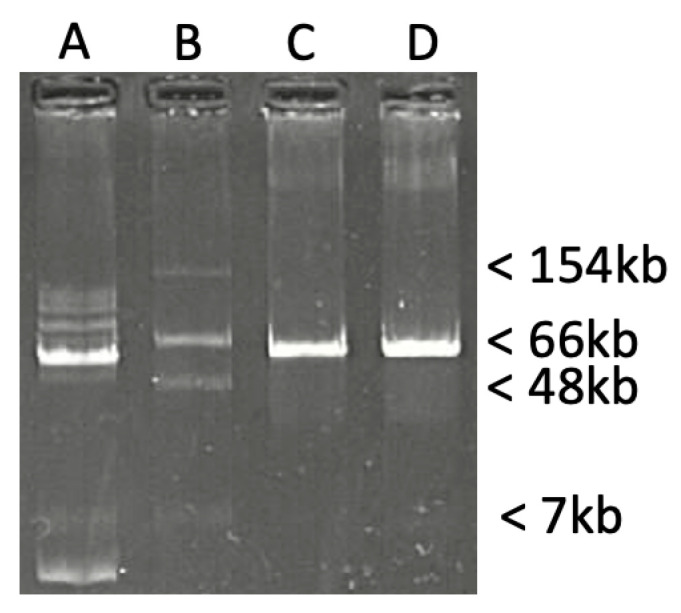
Kieser DNA extractions of (**A**) parental *E. coli* 172D10, (**B**) *E. coli NCTC* 50192 containing four plasmids of ca. 7-, 38-, 66-, and 154-kb, (**C**) *E. coli* TOP10 pN-OXA-484 electroporant, and (**D**) *E. coli* J53 pN-OXA-484 transconjugants.

The direct transfer of the β-lactam resistance marker *E. coli* 172D10 into *E. coli* J53 by mating-out experiments revealed the transfer of the 58.4-kb plasmid (Figure 1), confirming the self-transferable nature of plasmid pN-OXA-484. Similarly, electroporation of this plasmid into *E. coli* TOP10 yielded transformants resistant to β-lactams and reduced susceptibility to carbapenems (Table 1).

**Figure 2:**
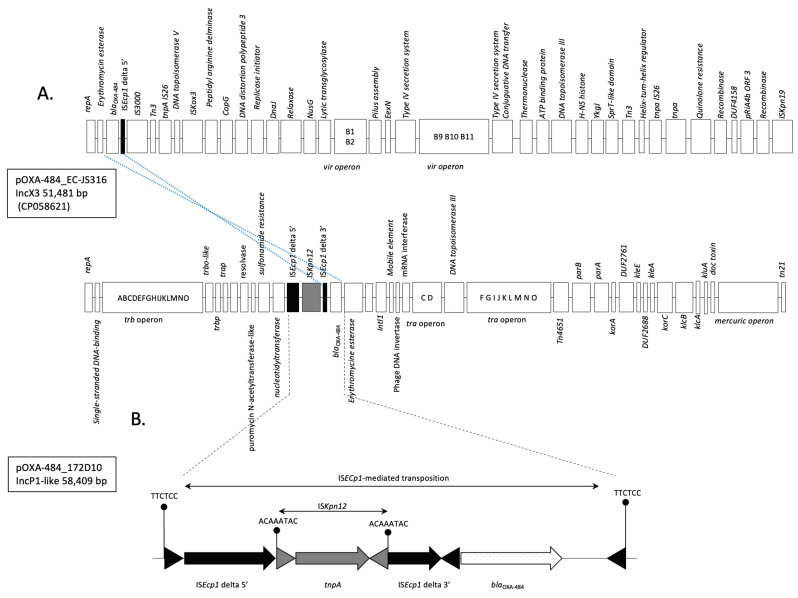
Schematic representation of (**A**) pOXA-484_EC-JS316 (IncX3 of 51,481 bp (CP058621) harboring *bla*_OXA-484_ and *qnr*S1 and pN-OXA484_172D10 (IncP1-like of 58.4-kb) harboring *bla*_OXA-484_ gene. Common structures are highlighted in dotted region lines. (**B**) Schematic representation of IS*Ecp1*—*bla*_OXA-484_ transposon in pOXA-484_172D10. Genes are represented by arrows. Target site duplications are indicated, and Inverted Repeat sequences are indicated by triangles. Colored-dotted boxes indicate resistance genes: *bla*_OXA-484_ and *qnr*S1. IS*Ecp1* and IS*Kpn12* insertion sequences are represented by black and grey boxes, respectively.

**Figure 3:**
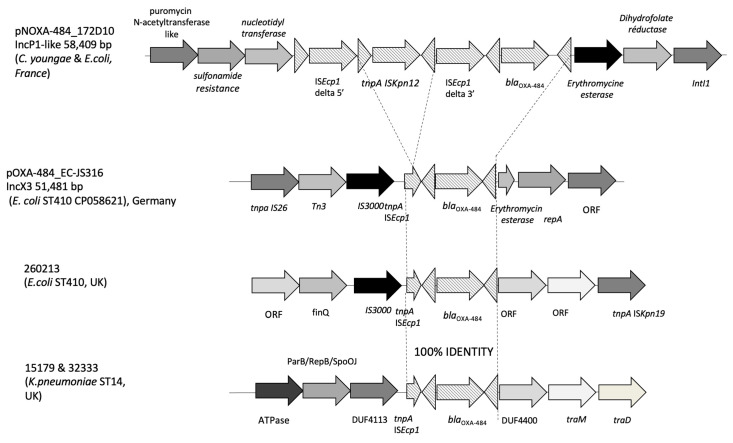
Structural features of the genetic environment of *bla*_OXA-484_ from pOXA-484_172D10 with pOXA-484_EC-JS316 (GenBank accession number CP058621 [9] and with three other strains from England [8,15]. Common structures are indicated by dotted lines.

According to PlasmidFinder, seven different plasmid replication origins belonging to the incompatibility groups IncB, IncN, IncP1-like, IncX4, col156, Col8282, and p0111 were identified in *E. coli* 172D10 and an IncP1-like one in *C youngae* 173G7 (Table 2). Plasmid reconstruction revealed that the *bla*_OXA-484_ gene was carried by an IncP1-type plasmid of ca. 58.4-kb in both isolates. This plasmid was different in size and in nature from previous plasmids described to contain *bla*_OXA-484_ genes, such as the IncX3 plasmid identified in *E. coli* ST410 from Germany, UK, and Switzerland (Figure 2 and Figure 3) [8,9,10,11,12,13,14,15]. The only part that was shared between these plasmids was the IS*Ecp1*-based composite transposon carrying *bla*_OXA-484_ gene (Figure 2B). This structure was inserted into the IncP1 backbone, as revealed by a target site duplication on both sides. In addition, in both isolates, the open reading frame of IS*Ecp1* was disrupted by the insertion of another insertion sequence, IS*Kpn12,* in contrast to what was observed for the IncX3 plasmids (Figure 3).

### 3.4. Biochemical Properties Determination

To characterize the impact of the R214G substitution in OXA-181 on the hydrolytic profile, steady-state kinetic parameters of OXA-484 for several clinically relevant substrates (penicillins, cephalosporins, and carbapenems) were determined and compared to those of OXA-48, OXA-244, OXA-181, and OXA-232 [3,4,6,7].

The *k*_cat_/*K*_m_ of OXA-484 for the hydrolysis of penicillin G, ampicillin, and cephalothin were like those of OXA-181 (Table 3), suggesting that the R214G substitution does alter the hydrolysis of these substrates. Interestingly, the hydrolysis of penicillin G was 61-fold lower with OXA-244. Oxyimino-cephalosporins such as cefotaxime were weakly hydrolyzed, but the *k*_cat_/*K*_m_ could not be determined precisely, as the *K*_m_ value was >1000 mM, determined experimentally. For ceftazidime, a bulkier oxyimino-cephalosporin, no hydrolysis was observed even with 1.92 µM purified enzyme and up to 500 µM substrate, as shown for OXA-48 [3,4].

Compared to OXA-181, OXA-484 had *k*_cat_/*K*_m_ values 3.3-, 7.5-, and 27.5-fold lower for ertapenem, meropenem, and imipenem hydrolysis, respectively (Table 3). The reduced catalytic efficiency for imipenem of OXA-484 is mainly due to a 75-fold reduction in the turnover number. As observed for OXA-232, the *k*_cat_/*K*_m_ for ertapenem of OXA-484 was 33.3-fold lower than that for imipenem but was not affected as compared to the value for OXA-181 or OXA-48 (0.6 s^−1^.mM^−1^ vs. 2 and 1 s^−1^.mM^−1^, respectively).

Regarding temocillin, the *K*_m_ was 3-fold higher than for OXA-181, showing that OXA-484 has a lower affinity for temocillin. Moreover, *k*_cat_ for temocillin of OXA-484 was 7.5-fold lower than that of OXA-181, thus resulting in a 21.7-fold lower catalytic efficiency for temocillin compared to OXA-181.

Determination of IC_50_ showed that OXA-484, OXA-232, and OXA-181 were similarly inhibited by clavulanic acid (19 µM, 13.4 µM, and 28.5 µM) and tazobactam (1.09 µM, 0.75 µM, and 20 µM), respectively.

Overall, the hydrolysis profiles were consistent with those of OXA-244. The catalytic efficiencies, *k_cat_/K_m_*, were concordant with the MIC results and confirm that the hydrolysis of imipenem depends on the nature of the residue in position 214, irrespective of the backbone of the enzyme.

### 3.5. Molecular Modeling

An in silico study was performed to identify the structural determinants that could explain the experimentally determined differences between the hydrolytic profiles of OXA-484 in comparison with OXA-181. The OXA-181 structure (PDB code 5OE0) was used to generate a model of OXA-484 by introducing in silico the mutation R214G.

**Figure 4:**
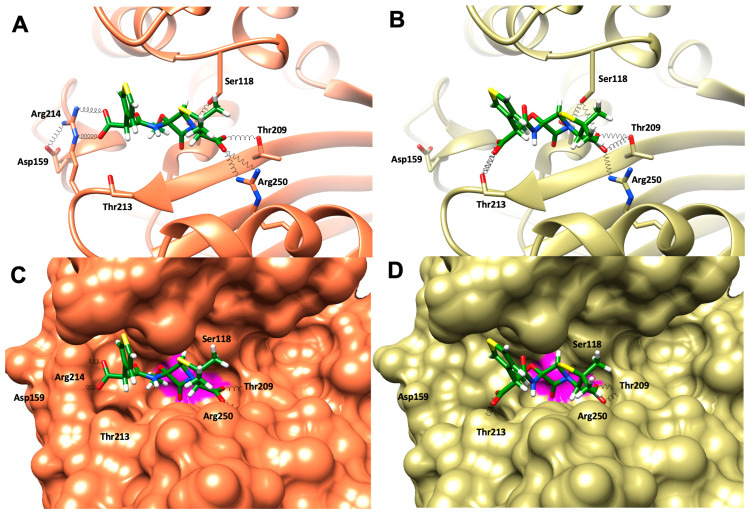
Crystal structure of OXA-181 (**A**,**C**, PDB code 5OE0) and in silico model of OXA-484 (**B**,**D**) in cartoon and surface representations, with the docking conformations of temocillin in stick representation. OXA-181, OXA-484, and temocillin are colored in orange, olive, and green, respectively. The surface of Ser70 is colored in magenta. Hydrogen bonds are represented as springs colored in black.

The salt bridge between D159 and R214 that was present in OXA-48 and OXA-181 was lost in OXA-484, similarly to OXA-244 [7]. The loss of this salt bridge resulted in significant changes in the shape of the active site at its periphery (Figure 4). In addition, as previously shown for OXA-48, R214 also established a favorable ionic interaction with the carboxylate group of the R1 substituent of temocillin, and this interaction was lost in OXA-244, which has a glycine residue in position 214 [6,7]. Here we show, through molecular docking calculations, that this interaction was also absent in the complex of temocillin with OXA-484, which has an OXA-181 backbone and a glycine in position 214 (Figure 4).

## 4. Discussion

OXA-48-producing Enterobacterales are now endemic in many countries and are increasingly isolated all over the world [1]. Along with the current spread of OXA-48, more than 60 variants have been described [5]. These variants can be classified into four groups according to their hydrolytic profile: (i) those that have an enzymatic activity similar to OXA-48, such as OXA-181 [36]; (ii) those that have no carbapenemase activity but, instead, a marked hydrolytic activity against ESC, similar to OXA-ESBLs, such as OXA-163, OXA-247, and OXA-405 [37]; (iii) those that possess carbapenemase and ESC hydrolytic activities, such as OXA-517 and OXA-438 [28,29]; and finally, those that exhibit an overall reduced activity towards all ß-lactams including carbapenems as compared to OXA-48, such as OXA-244 and OXA-232 [6,7]. The amino acid sequence comparison of OXA-48-variants suggests a link between the primary structure and the function of these enzymes. Indeed, all OXA-48-variants with an OXA-ESBL phenotype (loss of carbapenem hydrolysis and the gain of activity towards ESC) have an amino acid deletion in the β5-β6 loop, and those demonstrating both activities display a 2-amino-acid deletion [29,38]. This observation suggests that this loop plays a role in the substrate specificity. The phenotypic and enzymatic study of OXA-232 (which differs from OXA-181 by only one substitution R214S) [6,7] underlined that the residue 214 is crucial for carbapenem and temocillin hydrolysis. Similarly, phenotypic and enzymatic studies of OXA-244 (which differs from OXA-48 by only one substitution, R214G) [6,7] confirmed these results. The impact of the R214G substitution in an OXA-181 backbone was initially partially investigated by Oueslati et al., where enzymatic studies conducted on an in vitro generated mutant of OXA-232 (S214G) suggested that imipenem and ertapenem hydrolysis could be impaired in a similar manner to OXA-244 [6,7]. Here, detailed enzymatic activity of the OXA-484 variant with an R214G substitution in an OXA-181 backbone was analyzed.

From a treatment perspective, these enzymes, even though with reduced activity towards carbapenems, in a background of impaired outer membrane permeability, led to high-level carbapenem resistance. Unlike the *E. coli* ST410 isolates that were all multidrug-resistant [13], ST69 *E. coli* 172D10 was still susceptible to many classes of antibiotics. *E. coli* ST69 belongs to a dominant uropathogenic lineage frequently associated with AMR [39,40], while *C. youngae* ST491 is rarely described especially among CPEs [41].

Both 3D structures of OXA-48 and OXA-181 revealed the presence of a salt bridge between R214 and D159 [4,6] that maintains the shape and the network of water molecules within the binding site. Our molecular modeling study revealed that for OXA-48, a R214G substitution prevents any interaction with D159, which presumably increases the flexibility of this part of the binding site, thus confirming the critical role of the R214-D159 interaction in carbapenem and temocillin hydrolysis. This point is of utmost clinical importance and explains why the detection of OXA-244, OXA-232, and now OXA-484-producers remains a challenge for clinical microbiology laboratories [16,17,18]. Indeed, these isolates do not grow on ChromID Carba Smart (bioMérieux, Marcy L’Etoile, France), one of the most used media for CPE screening [16,17,42]. The ChromID Carba Smart is a biplate containing, on one side, a carbapenem and, on the other, temocillin, two substrates that are only weakly hydrolyzed by R214G variants of OXA-48/181 [16].

The *bla*_OXA-484_ sequence was initially reported in 2017 in five *Klebsiella pneumoniae* isolates from the UK [8]. In December 2019, it was described in an *E. coli* EC-JS316 strain belonging to ST410 obtained from a rectal swab of a patient hospitalized in Germany following a stay in India [9]. *Bla*_OXA-484_ was carried on an IncX3 plasmid of 51.5kb. Recently, *bla*_OXA-484_-ST410-*E. coli* isolates originating from India were described in Switzerland with both an IncX3 *bla*_OXA-484_-carrying plasmid and a core genome identical to EC-JS316 (23 ΔSNPs) [13]. This IncX3 plasmid is frequently described in *E. coli* ST410 strains harboring *bla*_OXA-181_ and *bla*_OXA-484_ genes isolated from patients but also from environmental sources. It has been suggested that an IS*26* composite transposon may underpin the mobilization of *bla*_OXA-484_ from this IncX3 plasmid to an IncFII plasmid found in *Klebsiella* in Switzerland and China and in *E. coli* ST1722 isolates from Switzerland [13]. Here, we observed a likely transfer of the transposon carrying *bla*_OXA-484_ to other plasmids, increasing the potential to spread to other *E. coli* strains and bacterial genera, such as *C. youngae*. Finally, *bla*_OXA_484_ gene has also been described on IncF-type plasmids in patient isolates of *K. pneumoniae* from the UK and environmental *K. pneumoniae* isolates from South Africa but also on an IncX3 plasmid in *K. variicola* from China [14]. In several cases, a link with India has been observed [8,9,10,11,12,13,14,15], which is not surprising given the high prevalence of *bla*_OXA-181_ genes carried by a common IncX3 plasmid in this country [1]. These data suggest that *bla*_OXA-484_ and *bla*_OXA-181_ genes may have evolved from a common ancestor and spread to different hosts and now to different plasmids, thanks to IS*Ecp1*, an element proficient in transposition at high frequency without a preferred insertion site and at the origin of *bla*_CTX-M-type_ ESBLs spread worldwide [43].

## 5. Conclusions

The importation of OXA-48-like producers from endemic areas to low-prevalence countries is a major concern, especially with difficult-to-detect mechanisms, such as those mediated by OXA-244 and OXA-484. This may lead to uncontrolled spread and the occurrence of outbreaks, as is already the case for OXA-244 [1,20]. Several countries have now reported OXA-484-producers from all the continents, suggesting active dispersion, likely because of under-detection and high mobility through transposition to various plasmid backbones. Therefore, infection prevention and control programs must be rigorously maintained and adapted to mitigate any future clinical impact due to these peculiar CPEs.

Detection of these enzymes is challenging, as they do not grow on all currently available screening media [16,17,42]. The mSuperCARBA™ medium has been shown highly efficient in the detection of OXA-244 producers, unlike the ChromID^®^ CARBA SMART medium [16,17,42]. The high prevalence of ESBLs among OXA-244 producers allowed for the detection of 77–78% of them using ESBL-specific screening media [16,17,42]. In our collection of isolates, three out of six expressed an ESBL and, thus, could also be isolated on these media. Molecular methods used for the detection of CPE carriers directly from rectal swabs may detect OXA-244 and OXA-484 producers [16,44], but subsequent plating on selective media may not yield any growth. Thus, microbiologists, in these situations, may consider using an ESBL-screening media or a different selective media to grow OXA-244, OXA-232, or OXA-484 producers [16,17,18]. Finally, CPE confirmatory tests should not be based only on biochemical assays such as the Carba NP, ß-Carba (BioRad, Marne La Coquette, France), or MBT STAR^®^-Carba (Bruker), especially in countries with high prevalence of these enzymes, but should rely on LFIA, such as the NG-Test CARBA 5 (NG Biotech) or molecular assays such as GenXpert (Cepheid, Sunnyvale, CA, USA) [16,23,44]. Carbapenem inactivation methods could be an interesting alternative, as the rCIM and CIM tests allowed detection of 7/8 (87.5%) and 6 out of 8 (75%) OXA-244 producers, respectively [45].

## Figures and Tables

**Table 1:** Antimicrobial susceptibility and confirmatory test results.

		MIC (mg/L) ^a^									
	*E. coli*	*C. youngae*		*E. coli* TOP10						*E. coli* HB4	
ß-lactam	172D10	173G7	pN-OXA-484	pTOPO-OXA-181	pTOPO-OXA-484	pTOPO-OXA-232	pTOPO-OXA-244	pTOPO-OXA-48	- ^b^	pN-OXA-484	- ^b^
Amoxicillin	>256	>256	>256	>256	96	>256	>256	>256	2	>256	16
Temocillin	64	128	128	>256	12	6	6	>256	4	>256	24
Cephalotin	ND	ND	ND	16	8	16	16	16	4	128	32
Cefotaxime	4	1.5	0.75	1	0.064	0.047	0.064	0.25	0.06	1.5	0.75
Ceftazidime	0.75	1.5	0.125	0.5	0.38	0.25	0.38	0.25	0.12	1	0.75
Ertapenem	1.5	1.5	0.75	0.125	0.047	0.047	0.064	0.094	0.003	>32	0.75
Imipenem	1.5	1.5	0.19	0.75	0.19	0.25	0.25	0.5	0.25	>32	0.125
Meropenem	0.75	0.75	0.125	0.094	0.016	0.016	0.047	0.047	0.016	>32	0.38
CarbaNP test ^c^	Neg	Pos/Neg	Neg	Pos	Neg	Neg	Neg	Pos	Neg	Neg	Neg
NG-test CARBA5 ^d^	OXA	OXA	OXA	OXA	OXA	OXA	OXA	OXA	Neg	OXA	Neg

^a^: values obtained with broth microdilution method; ^b^: values without plasmid; ^c^: Neg: negative test results, Pos: positive test result, Pos/Neg: inconsistent test results; ^d^: OXA: when the OXA-48-like band light up; Neg: when no band was visible after 15 min migration.

**Table 2:** Heatmap displaying the detected plasmid Inc-types, resistance genes, and the MLST of Enterobacterales harboring the *bla*_OXA-484_ gene.

Isolates				Plasmids														ß-Lactamases							Aminoglycosides									Fluoroquinolones					Others								
Bacteria	Number	MLST	Country ^1^	Size	IncP1	col156	Col8282	IncB/O/K/Z	IncR	IncFIB(K)	IncFIA	IncFIB	IncX3	IncY	IncN	IncX4	p0111	TEM-1	Shv-28	OXA-1	OXA-9	CTX-M-15	CMY-like ^2^	OXA-484	AadA1	aadA5	aph(6)-Id	aac(6′)-Ib-cr	aac(3′)-Iid	aac(3′)-Iia	aac(6′)-Ib3	aph(3”)-Ib	aac(6′)-Ib-cr	oqxA	oqxB	qnrS1	sul1	Sul2	Tet(B)	Tet(A)	ere(A)	mdf(A)	mph(A)	dfrA-^2^	fosA	cmlA1	Reference
*E. coli*	172D10	69	F	58.4																																								14 17			This study
*C. youngae*	173G7	491	F	58.4																			157																								This study
*K. pneumoniae*	32333	14	UK	ND																																								1			[8,15]
*K. pneumoniae*	15179	14	UK	ND																																								1			[8,15]
*E. coli*	260213	410	UK	ND																			42																								[8,15]
*E. coli*	EC-JS316 ^3^	410	G	51.5																																								17			[9]

^1^: Country of origin F: France, UK: United Kingdom, and G: Germany; ^2^: Filed boxes (light or dark grey) indicate presence of a gene, and numbers within boxes indicate the allele number of a given gene product. Boxes in dark grey represent the plasmid carrying *bla*_OXA-484_ gene. ^3^: Genbank accession number CP058621.

**Table 3:** Steady-state kinetic parameters for hydrolysis of ß-lactam substrates by OXA-48, OXA-181, OXA-232, OXA-484, and OXA-244.

	K_m_ (μM)					k_cat_ (s^−1^)					k_cat_/K_m_ (mM^−1^.s^−1^)				
Susbtrate	OXA-48	OXA-181	OXA-232	OXA-484	OXA-244	OXA-48	OXA-181	OXA-232	OXA-484	OXA-244	OXA-48	OXA-181	OXA-232	OXA-484	OXA-244
Benzylpenicillin	ND	90	60	33 ± 1.9	450 ± 37	ND	444	125	329 ± 8.4	72 ± 8	ND	5000	2100	9972 ± 724	163 ± 28
Ampicillin	400	170	220	653 ± 72.2	657 ± 163	955	218	132	394 ± 20.6	373 ± 158	2400	1300	600	604 ± 43	549 ± 133
Temocillin	45	60	60	178 ± 25.1	364 ± 23	0.3	0.3	0.03	0.04 ± 0	0.11± 0.02	6	5	0.5	0.23 ± 0.03	0.31 ± 0.05
Cefalotin	195	250	125	37 ± 2.6	88 ± 27	44	13	13	3 ± 0.2	3.3 ± 0.8	225	50	105	78 ± 1.7	39 ± 4
Ceftazidime	NH	NH	>1000	>1000	NH	NH	ND	>0.6	ND	NH	NH	ND	0.1	ND	NH
Cefotaxime	>900	>1000	>1000	>1000	>900	>9	>62	>6.5	ND	ND	10	13	6	ND	ND
Ertapenem	100	100	110	27 ± 1.2	22.9 ± 0.3	0.13	0.2	0.04	0.02 ± 0	0.02 ± 0.01	1	2	0.4	0.6 ± 0.06	0.1 ± 0.03
Imipenem	13	13	9	6 ± 2.1	2.6 ± 0.8	5	7.5	0.2	0.1 ± 0.01	0.07 ± 0.01	370	550	20	20 ± 4.4	27 ± 11
Meropenem	10	70	100	263 ± 9.4	23 ± 4	0.07	0.1	0.03	0.05 ± 0.05	0.02 ± 0.001	6	1.5	0.3	0.2 ± 0.06	0.8 ± 0.2

ND, not determinable; NH, no detectable hydrolysis was observed with 1.92 µM purified enzyme and up to 500 µM substrate. Data are the means of three independent experiments. Data for OXA-48, OXA-181, OXA-232, and OXA-244 were from Docquier et al. [4], Oueslati et al. [3], Oueslati et al. [6], and Rima et al. [7], respectively.

## Data Availability

WGS of *E. coli* 172D10 and *C. youngae* 173G7 were deposited under GenBank accession numbers JBAGCG000000000 and JBAGCF000000000, respectively (bioproject PRJNA1075029).
